# Collectanea

**Published:** 1828-11

**Authors:** 


### COLLECTANEA.

Floriferis ut apes in saltibus omnia libant,

Omnia nos, itidem, depascimur aurea dicta.

### ANATOMY.

*THE Uterus wanting.*—Dr. Breschet relates that, last February, a young woman, with fistula in ano, and who, from examination, appeared to have no uterus, applied to M. Dupuytren for admission into the Hôtel Dieu. She had never had the menses, yet she always experienced the symptoms which precede their periodical return. The pelvis was rather narrow, but the breasts and the external parts of generation were well developed, and her general appearance quite feminine. The vagina terminated, at about an inch from its orifice, in a cul de sac, smooth and circular, with no indications of a uterus. The rectum was explored, but it led to no discovery.

This female had been living several years in concubinage, and was to be married on her recovery from the fistula.

The operation was performed by M. Dupuytren on the 28th February last, and she died of acute hepatitis on the 15th of the following March.

The body was most carefully examined. The pleura, lungs, and liver presented various traces of inflammation; and the left kidney contained a fibrous cyst, full of a white and inodorous fluid. The clitoris and labia were well developed; but M. Dupuytren thought the cavity which occupied the natural situation of the vagina was the effect only of the efforts at coition. Above and behind the bladder were seen what appeared to be the broad ligaments of the uterus, in which were discovered eustachian tubes and ovaria of a large size. There was no matrix; but, where the tubes joined, their diameter was slightly augmented: yet this part had no cavity, and did not in the least resemble the uterus.—*Repert. d'Anat.*

### PATHOLOGY.

*Hypertrophia of the Brain.*—Hypertrophia of the brain has been latterly considered a primary disease of this organ; yet it has been, up to the present period, but vaguely described: and, indeed, the structural anomalies and pathognomic signs of this disorder are not to be understood nor explained without an attentive study of some well-defined cases. M. Dance has for this purpose adduced several very interesting ones; but he is properly careful to inform us, that he does not apply the term hypertrophia to an augmentation of the brain resulting either from inflammation of its substance, from serous or sanguine congestion there, or from effusion into its cavities. For the afflux and stagnation of the fluids may increase the apparent dimensions of this viscus, but are not incorporated or identified with its substance; whilst real hypertrophia, he maintains, essentially consists in an unnatural augmentation, either as to bulk or number, of the constituent molecules peculiar to each organ. Now the brain is liable to this excess of nutrition, and ultimately to acquire a volume disproportionate to the capacity of its osseous receptacle.

M. Dance details four cases, which are quite sufficient to prove the existence of this disease; but they are not numerous enough to furnish a complete history of this remarkable change of structure, though they tend considerably to improve our acquaintance with several of its distinguishing peculiarities. The following conclusions may be deduced from his cases.

1. Preternaturally increased nutrition of the brain is characterised by the flatness and approximation of its convolutions, the coarctation of its ventricles, and the unusual whiteness and firmness of these parts; and by a singular dryness of its parenchyma and of the arachnoid cavity; whilst the general texture of this viscus evinces no sensible alteration.

2. Hypertrophia has been repeatedly observed to predominate throughout the whole of the cerebrum, but never in the cerebellum.

3. Hypertrophia is so far from increasing the action and energy of the brain, that it decidedly tends to diminish, deteriorate, and suspend them: and these effects are no doubt owing to the continual pressure which the contents of the cranium are necessarily compelled to suffer, to a greater or less degree, in every instance of this ailment.

4. As the symptoms of hypertrophia have varied in different individuals, we are not yet prepared to define it with precision and correctness. However, this affection would seem to be developed very gradually, and under the influence of extremely occult causes.

The adult age may be considered a predisposing cause, (since, in all the four cases alluded to, the patients were between twenty and thirty years old;) and, as occasional ones, contusions of the head (Case 1st), and frequent determinations of blood to that part (Case 4th). But, though every one of these causes seem to be of an inflammatory kind, yet this affection appears to arise essentially from excess of nutrition. For, if we admit that inflammation is the cause, we must also admit that this identical inflammation has simultaneously invaded all the textures of the brain, and affected them all in the same manner and to the same degree, notwithstanding the different functions they perform, and their ultimate peculiarities of structure. But inflammation does not usually proceed in this manner. In a single organ, and especially in one so complicated as the brain, inflammation often produces at the same time congestion, ramollissement, suppuration, and induration. These considerations would induce the belief that hypertrophia is the effect of morbid nutrition; and if so, it is not difficult to conceive that the brain, submitted to an uniform stimulus of nutrition, must become eventually more dense in a similar uniform ratio.—*Ibid.*

*Acute Hydrocephalus, cured by the spontaneous Formation of an Abscess.*— June 20th, 1827, M. A. Larrey was reqnested to visit an infant, fourteen months old, who had appeared to be in great pain during the last five or six days, and had been treated for worms. Her pulse was strong and freqnent, her cries piercing, and she was comatose with convulsions. The breathing was easy, and the belly neither tense nor painful.—Six leeches were applied behind each ear; she was allowed nothing but milk, or water flavored with sugar and "eau de fleurs d'oranger."

On the 21st, the symptoms were aggravated, and she could keep nothing down; the evacuation of the urine and feces were suppressed; the lethargy lasted longer; the convulsions were more violent; the pupils were dilated; and the head was thrown backwards.—The head was enveloped in ice: sinapisms were applied to the legs, and a blister to the back of the neck, and, in the evening, another to the right arm.

On the 22d, there was no change.

On the 23d, the vomiting and convulsions were so much worse that, when they ceased for an instant, life seemed quite extinct. On this day M. Larrey was told that, twenty-five days before, the child had fallen upon her head. He applied a blister to the head, and the abdomen was rubbed with an embrocation of camphorated oil. The child willingly took the breast, but soon after vomited up the milk.

Excepting that a blister was applied to the left arm, nothing new occurred until the 28th. From that day to the 9th July she took eighteen grains of calomel, and had five drachms of mercurial ointment rubbed along the course of the spine, and behind the mastoid processes, but with no marked alteration, excepting that the vomiting and convulsions were not so frequent. This treatment, after a short intermission, was renewed again, but with no better success. After this, medicines were entirely omitted, for the patient appeared in the last stage of marasmus, and her death was hourly expected.

On the 31st July, however, (about two months after the fall,) an oblong tumor, of the size of a pigeon's egg, was observed at the inner and upper part of the left arm. The skin over it was slightly inflamed, and it was painful when touched. Cataplasms were immediately used, to hasten on its progress towards suppuration.

On the 2d August, about eight ounces of a white and very firm pus were evacuated from the tumor. The wound was appropriately dressed, and was completely cicatrised in five days.

The child seemed quite renovated from the very day the abscess was opened; and asked for meat, which she eagerly devoured, as she did indeed every kind of food which was offered to her. This appetite continued, without any bad effects, for ten days; and at the end of the month her health was quite restored. The little patient has remained well ever since, even during the period of teething.—*Journ. de la Soc. Roy. de Med. &c. de Toulouse,—Archives Generales de Medecine.*

The Parisian journalist expresses some doubts as to the exact nature of this disease; and we certainly find no evidence in the details which could persuade us that this was a genuine case of acute hydrocephalus.

### SURGERY.

*Case of Necrosis, with Destruction of right superior Maxillary Bone, and Regeneration of the Teeth.* By Dr. Krimer.—A boy, eleven years old, and of a feeble constitution, after being hastily cured of tinea, was immediately affected with painful swelling of the right superior maxillary bone. His teeth became loose, and pus in several places made its way through the gums. A probe could be passed into the antrum. The right nasal fossa was obstructed, and the eye of that side pushed upwards, so as to cause strabismus and diplopia. The canine tooth and first molar were extracted, which was followed by an abundant flow of pus. A tumor, situated above the internal angle of the eye, was likewise opened. The os unguis and nasal process exfoliated. After awhile, the eye resumed its natural situation, and the anomalies of vision disappeared. Seventy-two fragments of bone, weighing two drachms six grains, were gradually expelled from the ulcers of the gums. The teeth fell out; all the alveolar margin of the maxillary bone, the greater portion of its exterior and anterior surfaces, the base of the nasal fossa, the major part of the palate and nasal processes, and the os unguis, were all destroyed.

The disease increased during four months, when the ulcers began to heal. The patient's complexion became fresh and healthy, and his countenance resumed its former appearance.

The lad had continued well eight months, when he again complained of pain, with swelling of the gums, at the posterior part of the alveolar margin. Where an incision being made, it was attended with no traces of pus, but the pain subsided, and in a few days three molares were seen. Two months later, another molar tooth was discovered. The other teeth have not reappeared; but those which have been replaced have since augmented in size, and were in good preservation three years after their appearance.—*Bulletin des Sciences Medicales.*

---

*The good Effects of the Solution of Chloruret of the Oxide of Sodium upon Ulcers.* By M. Willaume, Surgeon in Chief of the Hospital of Instruction at Metz.—M. Willaume relates two cases in which he found the use of this solution very beneficial. The first was an ill-conditioned ulcer of the upper lip and ala of the nose. The second was a case in which numerous ulcers threatened to invade all the skin of the legs of an individual, who had been recently attacked with paroxysms of fever. Emollients had been tried without success, when chloruret of the oxide of sodium was applied, and speedily effected the cure.—*Ibid.*

---

*Case of Enlarged Bladder.* By John Bishop Estlin, Member of the Royal College of Surgeons, London, and of the Royal Medical Society, Edinburgh.

A gentleman, fifty-four years of age, consulted me in October 1827, in consequence of constant nausea and loss of appetite and strength. His tongue was foul, and his bowels confined. He informed me that for many months he has had some difficulty in passing his water; that a considerable quantity comes away in the day and night, but in small portions at a time, and often involuntarily and without any force. He assured me (and I place full reliance on the declaration,) that he had never laboured under gonorrhœa or any other form of venereal complaint.—I ordered him some cathartics, with calomel; and, when he visited me two days afterwards, he was somewhat better. I then prescribed for him an emetic and a bitter aperient infusion.

15th.—No better. Being anxious to ascertain the state of the urethra, I introduced a middle-sized bougie, which met with a degree of obstruction at six inches from the orifice, that moderate pressure could not overcome; and, as much pain was occasioned by the attempt, I desisted from it for the present.

18th.—I introduced a silver catheter, and found it pass into the bladder without any obstruction. A pint of urine was drawn off; a quantity much exceeding what he has passed at one time for many months.

It was my intention to have passed the catheter again, principally with the view of ascertaining if there were any calculus in the bladder, impeding the passage of the urine into the urethra; but the canal remained in a very uneasy state from the employment of the instrument yesterday; and, as he was under the necessity of going a journey on business in a day or two, I thought it better to delay the attempt.

30th.—He returned from his journey last night, in all respects worse. He has constant nausea, and he frequently passes urine involuntarily.—To have gr. xij. of Dover's powder each night.

November 2d.—Vomiting incessant. The quantity brought up from the stomach is far more abundant than the fluid he swallowed: the rejected matter is of dark colour and coffee-ground appearance. He has some slight alvine evacuations of similar fluid.—A few ounces of blood were drawn from the arm: it was buffy. No relief experienced from the bleeding. Calomel and opium were given yesterday, and are to be continued.

About the 3d, he directed my attention to a swelling in the abdomen, which had escaped my notice when I felt the epigastric region, and when I daily pressed the bowels to ascertain if any tenderness existed. I examined the tumor, and found it to be of an oblong form, situated in the right hypochondrium, about the outer edge of the rectus muscle, extending nearly from the eleventh rib to the right side of the symphysis pubis, and being particularly prominent about the situation of the inner abdominal ring. It somewhat distended the integuments, so as to be perceptible to the eye, and might be considered to be about three inches in width.

His account of this swelling was imperfect, but he believes that he first discovered it last week, while he was absent on his journey. I was unable to satisfy myself as to its nature. It did not answer to the description of any kind of hernia. It was not elastic, nor could any fluctuation be discovered: it seemed to possess considerable solidity. No inflammation existed, as pressure did not detect any tenderness; nor was there any unusual tension over the rest of the abdomen. Turpentine injections were administered, and cathartics and opium taken by the mouth. The stomach rejects every thing, and the bowels are but slightly evacuated.

6th.—He continued to get worse, and I was desirous of having another opinion on the case, and he was visited by my friend Mr. J. C. Swayne, surgeon, of Bristol. Upon an attentive examination, as far as we could come to any conclusion, the tumor appeared to be a mass of internal disease, agglutinating the contiguous parts, pressing upon the bladder, and impeding the action of the intestines. By both of us the patient's speedy dissolution was expected. To his friends and himself the same event appeared so certain that he made a final settlement of his affairs, with considerable effort. For the last two or three days, he has spoken as if he anticipated a fatal termination.—Small but frequent doses of cathartic extract, with opium and purgative injections, were ordered.

7th.—He becomes still worse: some delirium; urine continues to be evacuated, and there is no swelling immediately above the pubes. With the view, however, of exactly ascertaining the state of the bladder, and of assisting, by drawing off the water that might be there, the action of the bowels, we resolved upon introducing the catheter. So near did his death at this time appear to his friends, that they earnestly entreated he should be subjected to no further inconvenience, but allowed to have an undisturbed release. These objections were of course over-ruled, and I introduced the catheter. It passed without any difficulty, and a forcible flow of urine through it occurred. The tumor immediately began to subside, and by the time about three pints of water had been drawn off it entirely disappeared.

The general nature of the disease was now apparent. It could not be doubted that the tumor was a preternatural enlargement of the bladder, and it seemed most probable that the elongated part was the internal coat protruded through the muscular coat; in consequence of which, the natural efforts of the bladder to expel its contents forced them into this cavity, instead of overcoming the cause of resistance at the neck of the bladder. To what extent any morbid impediment existed at the neck of the bladder, it was not easy to determine. The catheter passed without obstruction, and examination per anum detected no disease of the prostate gland.

In a few hours, when the tumor began to form afresh, the urine was again drawn off: the vomiting lessened, and the pulse in the course of the day became firmer.

8th.—Vomiting less frequent; urine drawn off night and morning; the vesical tumor is formed some hours before the introduction of the catheter. Some feculent evacuation followed the enema.

9th.—Vomiting nearly ceased; feculent discharges after the enemas; no power of voiding the urine, but it flows involuntarily upon the re-appearance of the swelling. He takes nourishment.

From this time he gradually improved.

His convalescence was slow, but regular; and he is now (August 1828) returned to his usual state of health, excepting that he feels less strong than he was before his illness. He never allows the bladder to become so full as for any involuntary discharge to take place, or for the tumor to become perceptible. No voluntary power over the bladder has returned. Pain along the urethra is the indication of the necessity to introduce the catheter; and this generally occurs every five or six hours. He is able to walk about, and use his accustomed exercise.

It is probable that some of your readers may feel surprise that the nature of this gentleman's complaint was not sooner detected. Without any attempt to dispute their penetration, or to justify my own want of it, I give the case just as it occurred in practice, with the hope that it may prove useful to others. Late as the knowledge of the disease was obtained, it was a source of great satisfaction to me that it was procured in time to relieve the patient, instead of being discovered by a post-mortem examination; a period to which alone at one time I looked for an explanation of the symptoms.

When the nature of an obscure disease has been unravelled, there is often but little difficulty in deciding upon the course that should have been pursued: but they who have been longest accustomed to medical practice can best estimate the difficulties with which the path of the practitioner is beset in cases of an ambiguous kind, where a valuable life is at stake, and where the hopes, and fears, and interests of anxious relations are contributing to perplex his mind, and to increase his diffidence of his own judgment.—*Condensed from the Medical Gazette.*

---

*New Operations proposed for the Stone.*—The operation in common use at the Hôtel Dieu is the bilateral, a modification of that of Celsus, in which the prostate is cut obliquely downwards from the neck of the bladder on *both* sides. The incision in the prostate is thus twice the size of that in the ordinary lateral operation: it will therefore give exit to a larger stone, and render a smaller opening into the bladder necessary for this purpose. Yet cases have occurred where considerable effort has still been required for the extraction of large calculi, and where the death of the patient has been occasioned by consequent inflammation and suppuration within the pelvis. In these cases, notwithstanding the incision was made to its fullest extent in these two directions above named, *the gland was found to be lacerated in a stellated form.* It is presumed, then, that if two other incisions be made, one on each side obliquely upwards, the mischief of laceration may be averted.

Dr. Vidal, who suggests the quadrilateral incision, considers it to be a point of extreme importance not to cut beyond the margin of the gland into the bladder, and that the neglect of this precaution is the prolific source of those urinary fistulæ and suppurations which follow the operation.

Where the *incision is confined to the prostate,* and unaccompanied by laceration or contusion, the wounded portions of the gland, being swoln after the operation, are thereby brought into contact, and the urine, instead of escaping through the wound into the pelvis, passes through its natural channel. *Not so when the bladder has been wounded,* or when the opening has been made by lacerations or by the gorget, or when portions of the gland have been brought away by calculi studded by asperities on the surface.—*A Correspondent in Paris.*

---

*Another Operation for the Stone.*—Mention is also made of an operation by Balardini, entitled "*la taille mediano,*" or median incision, in the raphe of the perineum, extending from the bulb of the urethra to the sphincter ani. The bistoury of the operator is then passed into the bladder along the groove of the staff, and, by cutting its way out, divides the neck of the bladder, the prostate, and the membranous portion of the urethra. The operation is said to be effected with the greatest facility, and to be exempt from the numerous inconveniences attendant on other methods. As no vessels of consequence can be wounded, hemorrhagy may be certainly avoided. The rectum has never been cut in a single instance. The opening into the bladder is the shortest course that can be taken, and admits of greater dilatation than that which is made by any other method.

In comparison with the recto-vesical operation, it may be remarked, that, as no communication between the bladder and rectum takes place, the passage of urine into the intestine, or feces into the bladder, can never occur. This frequently happens in the recto-vesical operation, since, in thirty cases, five have preserved incurable fistulæ.—*Ibid.*

*Attempt to cure Hydrocele by the insertion of a Solution of Nitrate of Potash.* M. Dupuytren, having observed that a solution of nitrate of potass was readily taken up by serous surfaces, attempted to promote the absorption of the fluid in the tunica vaginalis, by mixing with it a solution of eighteen grains of nitrate of potass in water: an equal quantity of the vaginal fluid was withdrawn, to make room for the nitrous solution. The experiment did not succeed. Inflammation was excited, which formed no part of the intention of the operator; who, in answer to a question whether he expected a cure to be effected by adhesion of the vaginal coat to the testicle, replied in the negative; for nothing would be gained by employing new means of causing adhesive inflammation, inasmuch as we already possessed an effectual remedy in wine and water.

The production of inflammation in this case having been ascribed to the use of too large a dose of the salt, the experiment was repeated on one of M. Sanson's patients, with nine grains only. A month has elapsed without the slightest diminution in the tumor, excepting such as might be accounted for by the occasional oozing of fluid through the orifice.—*Ibid.*

---

*Convulsions cured by Ligature.*—A girl, between thirteen and fourteen years old, not having menstruated, had been subject for four or five months, without any known cause, to periodical attacks of convulsions. They began by acute pains in the extremity of the ring finger of the left hand, and which were succeeded by a feeling resembling the Aura Epileptica through the whole arm. The patient next lost her recollection, fell down, and had convulsions more or less violent, which left her in a state of great exhaustion, so that she recollected nothing that had happened. These attacks, which took place monthly, appearing to the physician to depend upon the want of menstruation, he directed his treatment accordingly; but at the same time he recommended a ligature to be placed round the finger in which the attack began, and by this means suspended the accession. The next day the same pain was felt, and the ligature was again applied; but, whether this was done too late, or was not sufficiently tight, the fit came on then. A fresh ligature was placed above the wrist, and the attack was cut short.

The patient, encouraged by this success, made use of this means whenever she felt the pain in her finger, and by so doing preserved herself from these attacks for several successive days, till the menstrual discharge appeared, and saved her from the risk of a relapse.—*Decadas de Med. et Chir. Pract.*

---

*Amputations of the Uterus.*—Since the last communication made by M. Lisfranc to the Academy of Medicine, he has performed seven amputations of the neck of the uterus. Of these seven patients, four are completely cured, and enjoy good health; two are under cure; and one is dead, in consequence of an attack of peritonitis, which followed the operation. He particularly mentions this fact, since it is the first instance of a patient dying of an affection of this kind; and the peritoneum could not in any way have been injured in the performance of the operation. M. Lisfranc has performed his forty-third amputation of the neck of the uterus, and has had only four unsuccessful cases.—*La Clinique.*

*Ligature of the external Iliac Artery, by* M. RICHERAND.—A man was brought to the Hospital St. Louis, with aneurism of the upper and anterior part of the left thigh, extending into the groin. It arose without any known cause. Its pulsations were so powerful, that fears were entertained of the rupture of the sac. The limb was tumefied, painful, and numb; his knee was bent, and the leg was incapable of being brought into a straight position.

The tumor was covered with ice for five days, and the patient bled. On the sixth, an incision, four inches in length, was made in the abdominal parietes, commencing about an inch and a half above the level of the anterior superior spinous process of the ileum, and terminating in the middle of the groin, so as to avoid the epigastric artery and spermatic cord. A small opening was now made in the peritoneum, just sufficient to admit the forefinger, by means of which the iliac vessels were separated. A flat blunt-pointed probe, pierced at the extremity, was used for the purpose of drawing out the artery; and a ligature, composed of two threads only, was passed round it, which the operator tied, after having previously ascertained that compression suspended the pulsation of the tumor. The pain in the limb instantly ceased. No constitutional disturbance followed the operation; not the slightest diminution of heat or sensibility. The patient considered himself well enough to leave his bed even on the day of the operation.

The tumor was reduced two-thirds on the thirtieth day of the operation, and is since quite cured.—*A Correspondent in Paris.*

---

*On the Danger from Hemorrhagy after the Extirpation of Internal Hemorrhoids.* By M. DUPUYTREN.—This distinguished surgeon asserts that "the operation should never be performed unless in cases where the life of the patient is in danger; and then, for the purpose of preventing consecutive hemorrhagy, the actual cautery should be applied to the arterial points from which the blood flows at the time of making the incision." Notwithstanding, however, this positive injunction, the actual cautery was not used in the only operation which I saw him perform; but no blood flowed from the incision, and the internal pile was small, although it occasioned indescribable anguish, probably from its being accompanied by fissure and spasmodic constriction of the sphincter ani.

In a clinical lecture founded on former cases, he thus expresses himself:

Hemorrhoidal tumors are of two kinds—external and internal. The external form a prominent ring surrounding the anus, composed of little tumors, varying in size and number, covered partly by the skin and partly by the mucous membrane of the rectum, and are more or less of a dark colour. This assemblage of tumors is known by the term *bourrelet hemorrhoidal externe.*

The internal, on the contrary, are formed above the sphincter ani, and are wholly covered by the mucous membrane of the rectum. They seldom appear externally, unless the patient has been long in an erect position, or after strong efforts to open the bowels. In such cases we perceive them forming in the centre of the preceding an internal hemorrhoid, called *bourrelet hemorrhoidal interne.*

The latter is much more dangerous than the former. This is the species that furnishes the copious discharges of blood which undermines the health of the patient, which degenerates in intractable and fatal disease of the rectum. It is this where excision so frequently produces internal hemorrhagy, and thereby occasions the loss of the patient.

It cannot, indeed, be denied that the removal of external piles is occasionally followed by hemorrhagy; but, as the flow is external, we have timely notice of it, and it is easily restrained. On the contrary, the excision of the internal tumor frequently produces a hemorrhagy within the bowels, often not to be detected until it has proceeded to a considerable extent; and the means hitherto employed have often failed.

To avert this mischief, the actual cautery should be applied at the time of the operation: but, supposing this precaution to have been neglected, or only to have been employed on one of the points of the incision, and hemorrhage should take place within the bowels, what are the signs by which it may be detected, and what are the means by which it may be remedied?

The patient labouring under internal hemorrhage first experiences a sense of heat, which seems to ascend the intestine. The pulse, at first intermittent and irregular, soon becomes imperceptible. Cold sweats, deadly paleness, and syncopes, more or less frequent, soon manifest themselves; and it is no longer possible to mistake the cause of the symptoms.

Under these circumstances, the patient should be instructed to make an effort to evacuate the blood, and to produce the descent of the intestine; to assist which, it may be useful to inject cold liquid. If the gut appear, the actual cautery is to be applied.

Is this (adds M. D.) a remedy without inconvenience? No. It occasions pain and inflammation, more or less acute; but these effects are transitory. The swelling may for several days impede the evacuation from the bowels; but, before the excision of the tumor, care should be taken to purge the patient, and keep him on low diet, to prevent the necessity of his going to stool.

M. D. closed his remarks by indicating the precautions necessary to be taken for the purpose of preventing the symptoms which often arise from the sudden suppression of the hemorrhoidal discharge.

The patients should be bled every two or three months. If the tumors were of the bleeding kind, leeches should be applied to the anus. If the secretion was purulent, it will be prudent to make an issue in some part of the body before the operation be performed.—*Ibid.*

---

*Extirpation of Cancers within the Rectum and Vagina.*—Among the operations deserving of notice, from the remarkable success resulting from their performance in the hands of the French surgeon, are extirpation of cancerous disease high up in the rectum and vagina. The unhappy victims of these complaints have hitherto been sacrificed to an opinion of the impracticability of an operation, from an opinion that the *materies morbi* had extended too deeply into the surrounding parts to admit of cure, even if the excision could be effected. M. Lisfranc ascertained, by pathological examinations, that the disease was fairly circumscribed, and generally confined to the mucous membrane; so that its removal might fairly be effected.

Six successful cases attest the propriety of the attempt. Three have been exhibited to the Academy of Medicine; a fourth, from her elevated station in life, would not submit to the ordeal. The fifth and sixth were recently performed, as follows:

Case V.—The woman was long affected with syphilitic protuberances in the rectum, for which she had ineffectually submitted to mercurial treatment in the Hôpital des Veneriens. Syphilitic treatment was again tried by M. Lisfranc, but the disease increased, and ulceration took place.

The performance of the operation having been determined, the lower part of the rectum was included between two semilunar incisions, which were carried through the sphincters. Then, by introducing the forefinger into the anus, the intestine was protruded to an extent of about two inches, and, by means of scissors curved on their flat surface, the whole of the cancerous mass was removed.

Case VI.—At the clinical lecture delivered on the 22d August at La Pitié, M. Lisfranc thus expresses himself:

At No. 17 of the ward St. Pierre, lies a woman, who some months since contracted a venereal disease, at the termination of which, protuberances appeared in the region of the anus, which resisted all treatment, local and general, and continued to increase until her admission into the hospital. At this period it was evident, on examination, that all the circumference of the rectum was affected; the anterior and posterior surfaces, to the extent of about two inches and a half laterally, somewhat less.

So extensive a diseased surface required serious attention. A few days were therefore suffered to elapse before it was determined to operate. The cancer, indeed, was far advanced, and threatened speedily the destruction of the patient. On the other hand, the extirpation was a measure of difficulty, and of such danger that it might be attended by fatal result during the operation. There was, however, a chance of success, inasmuch as it had proved effectual in five other cases.

On the fifth day the operation was performed, and on the sixth, when this lecture was delivered, no doubt existed of complete recovery.

Similunar incisions were made through the sphincters in the posterior part of the rectum, for the purpose of facilitating the protrusion of the diseased surface by means of the forefinger; but this was ineffectual, in consequence of the height to which it extended. He then cut through the side of the rectum longitudinally upwards. The effusion of blood was so copious that the plugging of the part with sponge for a few minutes became necessary. The diseased surface was then dissected from the continuous parts, beginning anteriorly. The operator kept two fingers of the left hand in the vagina, while assistants, provided with hooks, preserved the parts in a state of extension, so as to bring them as much as possible into view. The dissections posteriorly and laterally were less painful, and more expeditiously performed; but the operation, on the whole, was tedious and difficult.—*Ibid.*

---

*Amputation of Cancerous Penis discovered to be unnecessary, by an exploratory Practice of* M. Lisfranc.—The fact previously stated of the frequently circumscribed nature of cancerous affections enabled M. Lisfranc to preserve a patient from the very inconvenient process of amputating the penis, which had been deemed advisable. In this case, when brought into the theatre for amputation, by making a longitudinal incision through the whole of the diseased mass, until he arrived at the sound parts, he discovered that it was bounded inferiorly by the corpus cavernosum penis; so that its removal by a careful dissection was easily effected.—*Ibid.*

*Cancer of the Tongue removed by Incision and Ligature.*—In a case of cancer occupying the whole length of the tongue, a longitudinal incision was made on the right side of the diseased portion through its whole length, whereby the tongue was split into two parts, and a ligature being applied beyond the base of the disease, it sloughed away in a few days.—*Ibid.*

---

*Fatal Case of Ligature to Polypus Uteri.* By M. MARJOLIN, of the Hospital Beaujon.—A ligature was applied to polypus uteri, which for sixteen months previously had occasioned pains in the womb, extending to the loins, and accompanied by constant menstrual discharge. On the fifth day, acute pains supervened in the belly, especially in the left iliac region; the patient vomited, and had fever. The ligature was loosened, but in four days she died.

Instead of bleeding copiously, the only sanguineous evacuation produced was by forty leeches applied to the belly.

On dissection, pus was found in the vessels of the uterus, whose tissue was red. Five or six ounces of pus were contained in the peritoneal sac of the pelvis. The peritoneal covering of the uterus was inflamed and purulent.

Other fatal cases from ligature are recorded by M. HERVEY. M. DUPUYTREN uses the knife or scissors in preference.—*Ibid.*

---

*Nymphomania.*—At a meeting of the Royal Academy of Medicine, on the 1st of September, M. LISFRANC referred to a case of nymphomania cured by cauterization, and took occasion to remark that it was incorrect to consider all cases of nymphomania and hysteria as of a nervous nature. These affections are often dependent upon the inflammatory condition of the neck of the uterus, or on a turgescent state or hypertrophy of the body of that viscus. He related the case of a young lady who was affected with nymphomania, evidently the result of an inflammatory attack, and which was cured by antiphlogistic treatment, such as local bleedings, warm hip-baths, and emollient injections: these were retained in the vagina by means of a plug of charpie, and were renewed frequently. M. Lisfranc related about ten cases of these affections cured by the same means: nevertheless, he thinks that, when the inflammatory symptoms have been removed by proper means, cauterization may be beneficial.—*La Clinique.*

---

### MIDWIFERY.

*Emphysema following Labour.*—A young woman, of irritable temperament, was seized with peripneumony at the beginning of the eighth month of pregnancy. On the seventh day labour came on, and for more than four hours the pains were sharp. A little time after, an emphysematous tumor made its appearance at the upper part of the chest. A practitioner having been called in, found the patient in the following state: The head was of enormous size; the face and neck purple, and considerably swelled; the chest and limbs greatly exceeded their natural dimensions, and the swelling every where presented the characters of emphysema. The oppression was so great that suffocation seemed impending. A large bleeding from the arm was practised, and repeated in four hours; after which, the breathing was less laborious; at the same time the emphysema diminished, the head and face regaining their ordinary size and colour; but even then the patient could not lie on either side. As there was no lochial discharge, and the abdomen was very tender, eight leeches were applied to the vulva, and several bleedings from the arm had recourse to. The oppression is stated to have diminished under the use of these remedies, but the patient was much reduced; the tongue dry, the pulse frequent and small; the neck tumified to such an extent that the skin covering it was on a level with the face. A large sinapism was applied to the chest, and the tumified parts were covered with compresses dipped in aromatic wine. On the thirteenth day from the delivery, the state of the patient was rather more favorable ; but, as the abdomen was still tender, the leeches were repeated; at the same time, some soup and spoonsful of wine were administered. From this time the emphysema gradually disappeared, and the patient recovered.—*Decadas de Med. et Chir. Pract.*

